# Selection and reporting of usual care comparators when designing primary care trials of complex health interventions: a systematic review

**DOI:** 10.3399/BJGP.2024.0525

**Published:** 2025-08-27

**Authors:** Shoba Dawson, Katrina M Turner, Sarah Dawson, Tom Yardley, Alyson L Huntley

**Affiliations:** 1 Population Health Sciences, Bristol Medical School, University of Bristol, Bristol, UK

**Keywords:** comparator, feasibility studies, primary care, protocols, randomised controlled trials, usual care

## Abstract

**Background:**

Many primary care trials evaluating complex health interventions use a ‘usual care’ comparator. As usual care can vary across clinical sites, countries, and over time thereby affecting trial design and raising ethical considerations, attention should be given to its content prior to a trial starting.

**Aim:**

To understand how researchers select and describe usual care comparators when designing primary care trials of complex health interventions.

**Design and setting:**

A systematic review of primary care trial or feasibility study protocols undertaken worldwide.

**Method:**

Electronic databases were searched from 1 July 2020 until 20 June 2022.

**Results:**

A total of 83 protocols were included. A range of terms such as ‘usual care’ and ‘care as usual’ were used to describe usual care. Descriptions of usual care varied greatly in terms of the level of detail provided regarding selection and content, and were categorised according to the amount of detail they provided: basic (72%), moderate (16%), and comprehensive (12%). Few protocols justified the content of their usual care comparator, with most simply commenting that it was based on clinical guidelines or current practice.

**Conclusion:**

Different terms were used to describe usual care and most primary care researchers provided limited details on the selection and content of their usual care comparators when publishing study protocols. This has implications for transparency and replicability, and suggests that researchers continue to give limited attention to the content of usual care when designing their trials.

## How this fits in

Many primary care trials are pragmatic in nature and use usual care as a comparator arm to assess the effectiveness of new treatments or practices. It is known that usual care can vary between trial sites and practitioners, and that this variation can have methodological and ethical implications for a trial’s design. Researchers and reporting guidelines have emphasised the need for trials that use a usual care comparator to describe it in detail so it is clear what intervention is being evaluated, and to allow researchers and practitioners to consider the trial’s relevance to existing practices. This review highlights that there is marked variability and inadequate detail in the descriptions of usual care in primary care trial protocols. Future research should establish the most effective and efficient way of setting out the ‘usual care’ that is being delivered in practice, so that researchers can describe it, prior to a trial starting, when using it as a comparator.

## Background

Many primary care trials evaluating complex health interventions are pragmatic in nature and evaluate new or modified treatments or practices against a ‘usual care’ comparator. To ensure findings are relevant to clinical practice, the usual care comparator should replicate care given in everyday clinical practice.^1^ This sounds simple, but usual care can vary for the same condition, across clinical sites, countries, and over time.^2^ In addition, it is important that researchers designing trials know what usual care includes, as its content can affect methodological and ethical aspects of a trial — for example, the sample size required^3^ and whether care provided at different trial sites is an acceptable standard.^4^ The intensity and content of usual care could also affect between-group differences observed in a trial and, therefore, how effective the trial determines the intervention to be.^3^

The potential heterogeneity of usual care and its impact on a trial’s design means researchers should carefully consider its content when designing trials with usual care comparators, and document what it will include.^5^ Although some researchers will define usual care as including the full range of treatments available in practice,^6,7^ others have chosen to protocolise or restrict what usual care comprises when using it as a comparator arm.^8,9^ Reasons for protocolising usual care include wanting to protect trial participants or address variations across trial sites in terms of the quality of the care provided.^10^

Researchers have not consistently applied terminology when referring to usual care arms that include all treatments available in practice; for example, they may use terms such as ‘usual care’, ‘treatment as usual’, and ‘standard care’ interchangeably.^5^ When researchers have decided what their usual care comparator will include, they have used terms such as ‘protocolised’, ‘devised’, or ‘enhanced usual care’^5^ but, again, terms have been used interchangeably.

There is growing recognition of the importance of defining and describing usual care prior to a trial starting, and some reporting statements such as Consolidated Standards of Reporting Trials (CONSORT)^11^ and Template for Intervention Description and Replication (TIDieR)^12^ request that both intervention and comparator arms are detailed. Currently, it is not known to what extent researchers describe usual care comparators in protocols of primary care trials, why they use these comparator arms, and what terms they use to refer to them. Thus a systematic review was conducted to understand how researchers select and describe usual care comparators in primary care trials of complex health interventions.

Specific questions addressed were:

- How have researchers defined ‘usual care’, ‘standard care’, ‘treatment as usual’ (or other synonyms) in primary care trials?
- How have researchers reported and described usual care in trial and feasibility protocols?
- What information have authors used to select and justify their usual care comparator, and how have they gathered this information?

## Method

The protocol for this systematic review is registered with PROSPERO (reference: CRD42022347342) and the review methods and results were reported in accordance with the Preferred Reporting Items for Systematic Reviews and Meta-Analyses (PRISMA 2020) guidelines.^13^

### Search strategy and selection criteria

A comprehensive search strategy was developed and tested with support from an information specialist (Supplementary Box S1). Searches included Medical Subject Headings and free-text terms relating to usual care and synonyms, primary care, and randomised controlled trials (RCTs), including pilot and feasibility studies. The electronic bibliometric databases MEDLINE, Embase, the Cochrane Library, and PsycINFO were searched from 1 July 2020 to 20 June 2022 to capture recent practice and keep the review manageable within the timeframe available for the study. No language restrictions were applied, but an English-language abstract had to be available for initial screening. The inclusion and exclusion criteria are outlined in ^14^ Box 1.

**Box 1.** Inclusion and exclusion criteria

	Inclusion	Exclusion
Study types	Feasibility studies and protocols of primary care trials evaluating complex health interventions that include a usual care comparator arm, and feasibility and developmental studies informing the design of such trials	Articles reporting results of completed trials or any other study designs
Population	Any population in primary care	N/A
Intervention	Any healthcare provision in a primary care trial of a complex health intervention described as usual care (or synonym) that acts as a control or comparator arm. The Medical Research Council’s (2021)^103^ definition of complex interventions was used — namely, interventions that are complex due to their properties, such as the number of components involved, range of behaviours targeted, expertise and skills required to deliver/receive them, number of groups/settings/levels targeted, permitted level of flexibility of the intervention or its components	Trials of interventions evaluating medicines (for example, drugs or pills) that are focused only on treatment outcomes and not, for example, improving adherence
Comparator	The trial arm that is viewed as reflecting current practice — that is, usual care or standard care, or usual care plus intervention (enhanced usual care or boosted usual care)	Waitlist, drugs, or another intervention that does not represent usual clinical practice
Outcomes	How usual care (and its synonyms) is defined and described in primary care feasibility studies and trial protocols of complex health interventions to improve patient outcomes when used as a control/comparator arm What steps and information researchers have used to ascertain what their usual care comparator includes Any justification given for having a usual care comparator or what that trial arm includes	
Setting	Primary health care. The World Health Organization’s^104^ definition of primary health care was used — namely, *‘PHC* [primary health care] *is a whole-of-society approach to health that aims at ensuring the highest possible level of health and wellbeing and their equitable distribution by focusing on people’s needs and as early as possible along the continuum from health promotion and disease prevention to treatment, rehabilitation, and palliative care, and as close as feasible to people’s everyday environment.’* This definition was used as it is internationally recognised, but this review’s authors are aware it is very broad, so only studies that were undertaken in a primary care setting and/or involved primary care practitioners were included	Articles reporting trials undertaken in settings other than primary health care

### Screening

References were imported into Endnote and, after deduplication, they were imported into Rayyan.^14^ Titles and abstracts were independently assessed for inclusion by two reviewers using the predefined inclusion criteria shown in Box 1. The remaining study protocols were screened by one reviewer as inter-rater reliability was high (κ coefficient = 0.84). 50% of full texts were screened independently by the two reviewers and, because of high level of agreement (κ coefficient = 0.77), the remaining full texts were screened by one reviewer. Any conflicts were resolved through discussion with a third reviewer.

### Data extraction

A customised data extraction form was developed in Microsoft Word and tested on a random sample of five articles. The TiDieR checklist^12^ was consulted to identify important elements when reporting a trial arm, namely:

- rationale for the comparator arm;
- components of usual care included;
- who delivered it and how;
- where they delivered it; and
- frequency and duration of usual care.

Data were extracted by one reviewer and checked for accuracy by two reviewers. Discrepancies were resolved through discussion.

### Risk of bias

Risk of bias of the included evidence was not determined as the aim was to review trial protocols to understand how researchers select and describe usual care when designing trials and not to assess the quality of the protocols included.

### Data synthesis

Narrative synthesis using descriptive text and tables were used to summarise the data, and to identify similarities and differences within, and between, study protocols.^15^

### Categorisation of usual care descriptions

Using a similar approach as devised by Petersson *et al* ^16^ usual care descriptions were categorised as ‘basic’, ‘moderate’, or ‘comprehensive’, according to the amount of detail they provided:

- Basic — usual care described as ‘treatment as usual’ or ‘provided according to a clinical or practice guideline’, and/or with a simple list of the treatments/procedures/materials included. No information was given about what the treatment(s) entailed, delivery, dose, frequency, or duration;
- Moderate — featured some information about what included treatments involved, the provider, location, and/or dose/frequency/duration;
- Comprehensive — offered a detailed account of treatments/procedures/materials including provider, location, and dose/frequency/duration. Comprehensive descriptions sometimes also provided information on the mode of delivery.

The use of reporting guidelines was recorded in all three categories to assess whether they had influenced the reporting of usual care.

To aid and inform the definitions of these three categories, the descriptions that had been extracted were read and re-read to consider what information they provided and, therefore, what criteria or information should be used to decide whether they were, for example, basic or moderate. Descriptions of usual care were categorised independently by three reviewers. When there was uncertainty about how a description should be categorised, there was a team discussion and a ‘best fit’ was agreed.

### Patient and public involvement (PPI)

Prior to submitting an application for grant funding, the review was discussed with seven PPI contributors. All seven viewed the study as important, agreed with the proposed design, and suggested search terms for the review. In addition, one member of the group agreed to be a co-applicant on the grant. They commented on the review protocol, attended three team meetings, and is a named author of this article.

Upon review completion, a PPI meeting was conducted with five PPI contributors (including the co-author of this article) to share key findings and discuss how they should be disseminated. Group members stated that they understood the findings, but suggested visual methods (such as infographics) should be used when disseminating results to public audiences because individuals working outside of trials might struggle to understand findings that were simply summarised in writing.

## Results

### Overview of review process

6063 records were identified and, after deduplication, 4077 titles and abstracts were screened. In total, 293 study protocols were included for full-text screening and 83 were included in the review (Supplementary Figure 1).Although none of the study team were clinicians, the approach, search strategy, and findings were discussed with clinical colleagues working in primary care to explore their views on its applicability to general practice and future primary care research.

### Characteristics of included protocols

The 83 included study protocols were based in: the UK (*n* = 14),^17–30^ US (*n* = 11),^31–41^ Australia (*n* = 7),^42–48^ The Netherlands (*n* = 7),^49–55^ Canada (*n* = 4),^56–59^ China (*n* = 4),^60–63^ Spain (*n* = 4),^64–67^ Denmark (*n* = 2),^68,69^ Ethiopia (*n* = 2),^70,71^ Hong Kong (*n* = 2),^72,73^ Ireland (*n* = 2),^74,75^ Germany (*n* = 2),^76,77^ Pakistan (*n* = 2),^78,79^ Portugal (*n* = 2),^80,81^ Singapore (*n* = 2),^82,83^ Tanzania and Uganda (*n* = 1),^84^ Bangladesh (*n* = 1),^85^ Botswana (*n* = 1),^86^ Chile (*n* = 1),^87^ Europe (*n* = 1),^88^ France (*n* = 1),^89^ Guatemala (*n* = 1),^90^ India (*n* = 1),^91^ Mexico (*n* = 1),^92^ Nepal (*n* = 1),^93^ Norway (*n* = 1),^94^ Papua New Guinea (*n* = 1),^95^ Uganda (*n* = 1),^96^ South Africa (*n* = 1),^97^ Poland (*n* = 1),^98^ and Zambia (*n* = 1)^99^. The study protocols described pilot studies (*n* = 8),^18,27,47,72,74,78,84,96^ feasibility studies (*n* = 5)^17,20,29,56,70^, or full RCTs (*n* = 70).^19,21–26,28,30–46,48–55,57–69,71,73,75–77,79–83,85–95,97–99^

The study protocols detailed trials evaluating a wide range of healthcare interventions for mental and physical health (Supplementary Table S1). Overall, 55 of the 83 protocols described using reporting guidelines, as follows: Standard Protocol Items: Recommendations for Interventional Trials (SPIRIT) used on its own, or with CONSORT or TIDieR (*n* = 37), CONSORT (*n* = 13), TIDieR (*n* = 2), CONSORT and TIDieR (*n* = 1), CONSORT and Standards for Reporting of Diagnostic Accuracy (STARD or STARD-AI) studies (*n* = 1), and Consolidated Health Economic Evaluation Reporting Standards and guidelines from the Global Health Cost Consortium (*n* = 1).

### Usual care terms

A range of terms were used to describe usual care (Supplementary Table S2). Most of the included protocols used the term(s) ‘usual care’ or ‘care as usual’ (*n* = 36, 43%). Others used: ‘control arm’, ‘control group’, or ‘control condition’ (*n* = 10, 12%); ‘treatment as usual*’* (*n* = 9, 11%); ‘standard care’, ‘standard practice*’,* or ‘standard of care’ (*n* = 9, 11%); ‘routine care’ or ‘usual routine clinical care’ (*n* = 3, 4%); ‘standard vertical care’ (*n* = 1, 1%); and ‘referral as usual*’* (*n* = 1, 1%). The remaining protocols used context-specific terms, such as ‘enhanced care’ or ‘boosted care’ (*n* = 7, 8%), ‘usual primary care’ or ‘general practice care*’* (*n* = 2, 2%), ‘standard nutrition care’ (*n* = 2, 2%), ‘routine antenatal care’ (*n* = 2, 2%), and ‘usual physiotherapy’ (*n* = 1, 1%). When the terms ‘usual care’ or ‘routine care’ were used, it was apparent that, in most cases, they referred to existing standard practices. However, when authors referred to ‘treatment as usual’ or ‘standard care’, there was considerable variation in what the usual care comparator comprised, as it could include standard practice or another intervention. The terms ‘enhanced care’ or ‘boosted care’ were used to refer to standard practice, plus another intervention/treatment.

When analysing the data on evaluated interventions (Supplementary Table S1), there appeared to be no relationship between how well usual care had been described and: country of publication; whether the protocol detailed a full RCT, or a pilot or feasibility study; medical discipline; mode of delivery; or use of a reporting guideline.

### Usual care content descriptions

The authors categorised the protocols as basic, moderate, or comprehensive as per their methods (Supplementary Table S2).

#### Basic descriptions of usual care

In total, 60 study protocols were categorised as having a basic description of usual care,^17–22,24–34,36–43,45,47,48,51,55–58,60–62,65,66,68,71–77,80,81,85–89,92–98^ with four of these simply saying the comparator arm was ‘usual care’.^21,60,71,88^ These protocols usually provided a very brief description of what usual care entailed or gave a vague and broad description without further elaboration on usual care contents or how it was chosen. 38 of the 60 stated they had used a reporting guideline when writing the protocol.^18,21,22,26–30,33,36,37,42,43,47,48,56,57,60–62,66,71–75,77,80,81,85,87–89,92–95,98^

Some protocols reported that usual care had been defined according to practice guidelines, but they did not always reference the guidelines or detail what care was included.^74^ Other protocols reported that usual care was provided in accordance with usual practice in the region or based on national/international/condition-specific guidelines — however, again, the content of usual care was not described.^86,97^ Some protocols detailed what treatments or support participants would *not* receive, rather than detailing that which they *would* receive.^98^

Only one protocol acknowledged a variation in usual care delivered across sites.^81^

#### Moderate descriptions of usual care

Thirteen study protocols were categorised as having a moderate description of usual care.^23,35,44,52,53,63,69,70,79,83,90,91,99^ Typical examples included information on usual care, along with some information on who delivered it; in some cases, timing of follow-up and/or treatment duration was included. Eleven of the 13 protocols stated they had used a reporting guideline.^23,44,52,53,63,69,79,83,90,91,99^

Most protocols did not offer any justification for the content of usual care in this category. In one protocol, justification included the need to match the intervention group and maintain community trust.^35^ In some protocols, justifications included following relevant guidelines or basing the content of usual care on clinical guidelines and giving a description of usual care.^52,53^

#### Comprehensive descriptions of usual care

Ten study protocols were categorised as having a comprehensive description of usual care.^46,49,50,54,59,64,67,78,82,84^ Descriptions in this category included details of the treatment provider, location, duration, type of treatment, frequency, and follow-up. Six of the 10 protocols stated that a reporting guideline had been used.^49,54,64,67,78,82^

Justification for the content of usual care comparators included following the same care provided in the region/clinical practice guideline for the condition.^49^

## Discussion

### Summary

Researchers designing primary care trials used a range of terms to refer to their usual care comparator. Irrespective of the term used, the content of usual care comparators ranged from standard practice to a single intervention that the researchers had chosen to represent usual care. When usual care had been enhanced, this was sometimes reflected in the term used, but this was not always the case.

The majority (72%) of the included protocols gave only a basic description of usual care. There was little evidence of researchers establishing what usual care included prior to starting a trial, or ensuring that it would be similar across trial sites or met the standards outlined in clinical guidelines. A small number of studies provided a justification for the content of their usual care comparator, but most simply stated that it was based on local/national clinical guidance or current practice. Although researchers stated that they had used reporting guidelines, there was little evidence that such guidelines had resulted in detailed descriptions of the usual care arm.

### Strengths and limitations

Databases were searched from 1 July 2020 to 20 June 2022, thereby limiting the timeframe of the review but including protocols that were published during the COVID-19 pandemic. This 2-year timeframe meant that recent practice was reviewed and, although the pandemic will have affected how trials were delivered during this period, there was no evidence in any of the included protocols that it had affected how usual care had been selected and described in the trials detailed.

Some of the included protocols were difficult to assign to the categories developed, and their allocation was subjective; however, this allowed for gauging what proportion of protocols only provided a basic description and what proportion described usual care in a way that enabled the reader to know what it included and how it was delivered.

Limitations were that only studies published in English were included. As the term ‘usual care’ was not used consistently, some relevant articles may have been excluded. However, the search strategy was designed with input from an information scientist, a researcher experienced in trial methodology, a PPI co-applicant, and other trialists.

### Comparison with existing literature

Other works have commented on researchers’ limited descriptions of usual care comparators in different settings.^16,100,101^ Given that a usual care arm has a critical role to play in ethical and methodological aspects of a trial, it is surprising that there has been minimal consideration given to its content. This could be because comprehension of trials is constrained by the notion that the experimental arm is the intervention to focus on, while the usual care control is merely a necessary framework for the trial.^5,102^

### Implications for research and practice

This descriptive systematic review is a sister publication to a methodology review that aimed to summarise current thinking on what should inform the content of usual care comparators.^5^ Various drivers have been identified that should inform this decision, including establishing, prior to a trial starting, the actual care being provided in practice. This will take time and resources, and given the potential heterogeneity of usual care, defining it could be challenging.

Establishing the most effective way to do this is still unknown, but many trialists conduct feasibility studies prior to a main trial; such studies could allow researchers to establish what usual care includes. In addition, with the development of NHS Digital, and the potential for trials and routinely collected NHS data to become more integrated than at present, there are opportunities to understand usual care in greater depth. Furthermore, it might also be possible to analyse routinely collected electronic health record patient data via, for example, the Clinical Practice Research Datalink (CPRD) database. CPRD collects these data from UK-based GP practices and provides patient-level information on various aspects of care, such as the administrative nature of consultations (frequency, length, staff member, continuity of care), diagnosis, investigations, medications prescribed, other clinical interventions (for example, reviews), and referrals made.

Primary care researchers use a range of terms to refer to their usual care comparator. Usual care remains poorly described in protocols of primary care trials, despite recommendations from reporting guidelines and a growing recognition of the need to do so. In addition, researchers provide limited justification for the content of their usual care comparator; most simply indicated it was based on local/national guidelines or current practice. These issues have implications for transparency, replicability, and reproducibility. Journal editors and reviewers should make it a requirement that reporting guidelines are followed, and researchers should be encouraged to view usual care comparators as complex interventions that will affect how a trial is designed and interpreted, and therefore be fully considered and documented before a trial starts. Uncertainty remains about how best to decide on what should form the content of a usual care comparator, but the recent methodology review indicated what might drive its content and what steps researchers could take to inform their decision.^5^

## Data

All data presented in this article are from publicly available papers as they are all published. Extracted data are available as tables in the manuscript.
